# Cancer incidence patterns by region and socioeconomic deprivation in teenagers and young adults in England

**DOI:** 10.1038/sj.bjc.6603794

**Published:** 2007-05-15

**Authors:** R D Alston, S Rowan, T O B Eden, A Moran, J M Birch

**Affiliations:** 1 Cancer Research UK Paediatric and Familial Cancer Research Group, Royal Manchester Children's Hospital, Stancliffe, Hospital Road, Manchester M27 4HA, UK; 2 National Cancer Intelligence Centre, Office for National Statistics, 1 Drummond Gate, London SW1V 2QQ, UK; 3 Teenage cancer Trust Young Oncology Unit, Christie Hospital NHS Trust, Withington, Manchester M20 4BX, UK; 4 North West Cancer Intelligence Service, Christie Hospital, Withington, Manchester M20 4BX, UK

**Keywords:** teenage and young adult cancer, geographical incidence, socioeconomic deprivation, cancer registration

## Abstract

Data on 35 291 individuals with cancer, aged 13–24 years, in England from 1979 to 2001 were analysed by region and socio-economic deprivation of census ward of residence, as measured by the Townsend deprivation index. The incidence of leukaemia, lymphoma, central nervous system tumours, soft tissue sarcomas, gonadal germ cell tumours, melanoma and carcinomas varied by region (*P*<0.01, all groups) but bone tumour incidence did not. Lymphomas, central nervous system tumours and gonadal germ cell tumours all had higher incidence in less deprived census wards (*P*<0.01), while chronic myeloid leukaemia and carcinoma of the cervix had higher incidence in more deprived wards (*P*<0.01). In the least deprived wards, melanoma incidence was nearly twice that in the most deprived, but this trend varied between regions (*P*<0.001). These cancer incidence patterns differ from those seen in both children and older adults and have implications for aetiology and prevention.

Cancer affects approximately one-third of the United Kingdom (UK) population before their 75th birthday and accounts for about 1 in 4 deaths (Coleman *et al*, 1999). Although predominantly a disease of old age, cancer in teenagers and young adults (TYAs) is an appreciable health problem and the commonest disease-related cause of death at ages 15–24 years in England (Office for National Statistics, 2002). Cancers in later adulthood are often associated with environmental or lifestyle risk factors and can be ascribed to the cumulative effect of exposures over a long time period. Chronic environmental exposures are thought to be less important in TYA cancers and genetic factors may play a greater role, although little is known about their relative importance (Birch, 2005).

Descriptive epidemiological studies indicate how disease incidence varies by demographic group and over geographic region and time. Such studies can provide clues to aetiology and disease prevention. Geographical variability in the incidence of childhood cancer has been studied extensively (COMARE, 2006), but cancer patterns in TYAs have been relatively neglected. National cancer registration in England provides reliable population-based incidence data over a long period, with substantial numbers even for rare cancers (Quinn *et al*, 2001). We have used national data to investigate variations in TYA cancer incidence by geographic region and economic deprivation for specific diagnostic groups.

## METHODS

Incidence data on all registered neoplasms diagnosed in 13–24 year olds in England from 1979 to 2001 inclusive were supplied by the National Cancer Intelligence Centre, Office for National Statistics, London (ONS). Data items included date of birth, age and year of diagnosis, sex, and coded diagnosis. Cases diagnosed from 1979 to 1994 were allocated International Classification of Diseases (ICD), Ninth Revision, disease codes and International Classification of Diseases for Oncology (ICD-O), First Edition, morphology codes (World Health Organisation, 1975, 1976). Cases from 1995 to 2001 were allocated ICD Tenth Revision disease codes and ICD-O, Second Edition morphology codes (Percy *et al*, 1990; World Health Organisation, 1992). Cancers were grouped according to the diagnostic scheme described by Birch *et al* (2002). Non-melanoma skin cancers were excluded but benign intracranial and intraspinal neoplasms were included.

The Government Office Region (GOR) and Townsend deprivation index (TDI) (Townsend *et al*, 1988) for the census ward of residence of the case at the time of diagnosis were also supplied. Cases from 1979 to 1995 inclusive had 1991 census ward TDIs and the 1996–2001 had 2001 census ward TDIs. The four components of the TDI are the proportion of households in the census ward that: have an unemployed economically active head; have no access to a car or van; are overcrowded are not owner-occupied. These components and resident populations by census ward for the 1991 and 2001 censuses were obtained from the national censuses (Office of Population Censuses and Surveys, 1991; Office for National Statistics, 2001). Annual population estimates for England by sex and single year of age and by sex and five year age groups for each of the nine GORs were obtained from the Population Estimates Unit of ONS.

The underlying population and number of cases in each cancer group were tabulated by time period, age group, sex and GOR. The periods were 1979–84, 1985–89, 1990–95 and 1996–2001 and the age groups were 13–14, 15–19 and 20–24 years. These populations and case counts were used to calculate observed and expected incidence and rates for census wards and GORs. The incidence rates by GOR were standardised to the European standard population using the direct method (Quinn *et al*, 2005). The relationship with TDI was analysed by grouping census wards into quintiles so the expected incidence for all cancers across England was the same in each quintile. For each cancer group and quintile, the percentage observed over expected (percentage risk ratio) was calculated and the significance of the trend and heterogeneity in risk after taking into account the trend was assessed using Poisson regression (McCullagh and Nelder, 1989). Poisson regression was used to compare the observed and expected incidence by GOR. Additionally, the incidence rates by GOR and TDI quintile were examined for variation by GOR after taking into account the trend by TDI (*P*-value after TDI) and to see if the trend in TDI varied by GOR.

## RESULTS

There were 35 291 cases occurring during 186 million person years at risk (mpyr), yielding an overall incidence rate of 188 cases per mpyr (Table 1). All rates given in the text are cases per mpyr. Incidence was slightly higher in males than females and greater in older individuals, but these patterns were not consistent across cancer groups. Across all cancers there was a statistically significant trend towards lower rates with increasing deprivation (Table 2a). After adjustment for TDI there was statistically significant variation in incidence by GOR, with the highest rate in the South East and lowest in the North East (Table 2b). However, incidence patterns by TDI and GOR varied by diagnostic group.

### Leukaemia and lymphoma

Leukaemia incidence overall was highest in the most deprived quintile, particularly for chronic myeloid leukaemia (CML), but there were no significant trends for acute lymphoblastic leukaemia (ALL) and myeloid (AML). Highest rates overall were seen in London and the South East, but rates for ALL did not vary significantly by GOR (Table 2b).

Lymphoma incidence showed a marked trend towards lower rates with increasing deprivation, entirely due to Hodgkin lymphoma (HL). However, both HL and non-Hodgkin lymphoma (NHL) incidence varied by GOR, with higher rates in regions in the South than in the Midlands and North (Table 2a and b).

### CNS tumours

Overall incidence of CNS tumours varied by TD1 (Table 3a), with higher rates in less deprived census wards, but the difference was only significant for unspecified CNS tumours. Rates varied significantly by GOR for astrocytomas, other gliomas, other specified tumours and unspecified tumours (Table 3b). A greater proportion of registrations from the West Midlands had unspecified morphology, 26% compared with 5% in other regions, and also had the lowest incidence among specified tumour groups except medulloblastoma. However, the inter-region variability in incidence of CNS tumours was still significant when the West Midlands was omitted from the analyses (*P*=<0.0001).

### Bone and soft tissue sarcoma

Incidence of bone tumours was unrelated to TDI (Table 4a) and GOR (Table 4b), overall and for osteosarcoma, chondrosarcoma and Ewings tumours. The ‘other bone tumours’ group showed considerable variability, incidence being highest in the West Midlands, again, due to excess cases with unspecified morphology.

There was no evidence of variability in incidence of soft tissue sarcomas (STS) by TDI (Table 4a). Incidence varied significantly by GOR (Table 4b), with the highest rate in London and the lowest rate in the North West, due mainly to ‘other specified STS’. This group includes synovial sarcoma, liposarcoma, leiomyosarcoma and other rare types, but case numbers were too small for firm inference about which types were causing the effect.

### Germ cell tumours and melanoma

There was a significant trend in the incidence of germ cell tumours (GCT) by TDI (Table 5a), with highest rates in the least deprived quintile. This was entirely due to testicular tumours, which formed 85% of GCTs. Incidence varied by GOR with highest rates in the South West.

**Figure 1:**
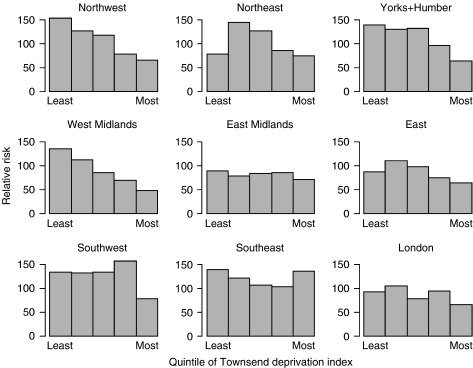
Percentage relative risk of melanoma in 13–24 year olds, 1979–2001, by Government Office Region and TDI using common quintile boundaries across England.

Melanoma incidence varied markedly by TDI (Table 5a). Highest rates were in the least deprived quintile and were almost double those in the most deprived. Rates varied significantly by GOR (Table 5b). Further examination of the data demonstrated that the relationship with TDI differed by GOR (Figure 1), with a uniformly high rate in the South West (*P*=0.85 for trend), uniformly low rates in the East Midlands (*P*=0.67) and East of England (*P*=0.31) and a striking trend in incidence with TDI in the North West, West Midlands and Yorkshire and the Humber (all *P*⩽0.0001). For these three regions, incidence rates in wards in the least deprived quintile were more than twice those in the most deprived and comparable with the overall rate in the South West.

### Carcinomas

Overall, there was no variation in incidence of carcinomas by TDI, but the incidence of carcinoma of cervix showed significant variability, with higher rates in the most deprived quintiles (Table 6a). There was a less marked trend in the opposite direction for breast cancer. Rates for lung, breast, cervix and colorectal carcinomas varied by GOR particularly for the latter two sites (Table 6b). Highest rates for lung cancer were seen in the South West, where the incidence was five times that in the East of England, although overall rates were low.

## DISCUSSION

This is the first study of variability in cancer incidence by region and deprivation score for TYA cancer patients in England and makes use of information from all nine regional cancer registries over a 23-year period. Data collection methods differ between the regional registries and some differences in ascertainment may exist. However, a study of registration of childhood cancers (aged 0–14 years) by the regional cancer registries estimated that under-ascertainment was less than 5% (Hawkins and Swerdlow, 1992). Ascertainment of cancers in young people aged 15–24 years is also likely to be high. Overall, cancer registration data from the 1970s onwards are mainly complete and of high quality (Office for National Statistics, 2006). It seems reasonable to suppose, since different cancer groups show different patterns by region and deprivation, that these results cannot be explained by differences in registration practices alone.

Large-scale variability in the incidence of neoplasms at all ages has been studied for the UK and Ireland (Quinn *et al*, 2005), and in relation to economic deprivation (Quinn *et al*, 2001, 2005). Since more than 75% of cancers occur over the age of 60 years in the UK, disease patterns in the young are masked by the much greater cancer incidence at older ages. Furthermore, these earlier studies grouped cases by ICD, which uses mainly primary site to classify cancers. As such a classification is inappropriate to cancers in the TYA age group, we developed a more suitable morphology-based classification (Birch *et al*, 2002) and this has been applied here.

Leukaemia incidence at all ages (Quinn *et al*, 2005) and also in childhood in Great Britain, 1969–1993 (COMARE, 2006), showed higher rates in areas of higher socioeconomic status, much stronger below age 10 than in 10–14 year olds. Our results show a slight trend in the opposite direction, with lower incidence in TYAs in more prosperous areas. The markedly higher incidence of CML in more deprived areas is a new finding, which should be verified in an independent study. Variability in incidence patterns between age groups may imply differences in aetiology. There is consistent evidence of a role for infections in leukaemia in young children (McNally and Parker, 2006), but other factors such as traffic density (Nordlinger and Järvholm, 1997) and benzene exposure (Schnatter *et al*, 2005) should be explored in TYAs, especially in densely populated areas with high leukaemia incidence, such as London and the South East (Table 2b).

Alexander *et al* (1991) found that HL incidence among 0–24 year olds in parts of England and Wales was greater in areas of high socio-economic status, and we confirm this for TYAs over a longer period and larger geographic area. HL in early childhood is related to Epstein Barr virus (EBV) infections, but in TYAs the predominant subtype is nodular sclerosis, which is associated with a much lower incidence of EBV inclusion within the tumour (Jarrett *et al*, 1996). HL in older childhood has been related to delayed exposure to infection arising from improved socioeconomic conditions (Glaser *et al*, 1997). Law *et al* (2003) found marginal evidence for higher NHL incidence in under-15 year olds in less deprived areas in England and Wales, but was not found here. Viruses are implicated in the aetiology of NHL, specifically with HIV l and HTLV l, as well as EBV (Baris and Zahm, 2001). Geographical and socioeconomic variations may reflect differing opportunities for exposure to relevant infectious agents.

McKinney *et al* (1994) found that incidence of childhood brain tumours in Scotland varied by geographic region, with an excess in more prosperous areas. This pattern is present in our TYA data and is also seen across all ages (Quinn *et al*, 2005), and in childhood CNS tumours (COMARE, 2006). Regional trends in specific types are difficult to analyse due to the large proportion of unspecified cases from the West Midlands and changes in proportions with specified morphologies over time.

In this study, we found little or no variation in incidence of the majority of bone and soft tissue sarcomas with geographical region and TDI. A comparable lack of variability in incidence was found in the childhood cancer study (COMARE, 2006). These results imply that aetiological agents are acting uniformly across geographical regions and socioeconomic groups, perhaps related to intrinsic factors affecting growth and development and genetic susceptibility (Birch, 2005).

In TYAs, GCTs are dominated by testicular teratomas and seminomas. The geographical pattern of testicular GCTs in the present study is similar to that for testicular cancer of all ages (Quinn *et al*, 2005), in keeping with most being GCTs, with highest rates below age 50 years. That study, like the present, also found higher rates in the least deprived groups. There is strong evidence that testicular GCTs have their origin during prenatal life and may be associated with maternal exposures during pregnancy (Møller and Evans, 2003), but how such factors relate to observed patterns is unknown.

In the UK, approximately 40% of melanomas occur under 50 years of age. The regional variations among TYAs largely reflect those reported for all ages (Quinn *et al*, 2005), with high rates in the South West and South East and low rates in London, East Midlands and West Midlands. Both studies also found a marked inverse association with socioeconomic deprivation. However, the restriction in the trend in incidence by TDI at ages 13–24 to certain regions of England is a new and striking finding. It is well documented that the greatest risk factor for melanoma is excessive exposure to ultraviolet radiation, especially during childhood. Light-skinned individuals are more vulnerable (Tucker and Goldstein, 2003), and regional differences in darker skinned ethnic minority populations may contribute to differences in rates, and to regional differences in the incidence gradient with TDI. Differences in reporting practices for melanoma between UK cancer registries may account for some but not all regional differences in rates (Goodwin *et al*, 2004).

The South West of England has the lowest lung cancer incidence and lower smoking levels than other parts of the UK (Rickards *et al*, 2004; Quinn *et al*, 2005). However, it also has high concentrations of residential radon (Green *et al*, 2002), which has been estimated to increase overall lung cancer risk by 8% per 100 Bq m^−3^ (Darby *et al*, 2005). The comparatively high rate of lung cancer and uniformity of incidence by TDI quintile in TYAs is in marked contrast to all ages data (Quinn *et al*, 2005). This would support the view that tobacco is not an important cause of lung cancer in TYAs, among whom radon exposure may be a factor.

Carcinoma of the cervix is the most common carcinoma in female TYAs in England. In the general adult population, this is related to sexual behaviour and human papilloma virus infection (Wallbloomers *et al*, 1999), with a much higher incidence in areas of high deprivation and in the North West, Northern and Yorkshire health areas (Quinn *et al*, 2005). Our similar results imply that aetiology in TYAs may be similar to that in older women.

Although incidence of colorectal carcinoma in most regions was close to the overall rate of 3.1, there was more than a twofold difference between regions with the highest and lowest rates. At older ages, dietary factors affect colorectal cancer risk (Norat *et al*, 2005), but whether this relates to regional differences in incidence among TYAs is unknown.

Cancer represents a major source of morbidity and mortality in 13–24 year olds. The patterns of incidence observed in the present study merit further investigation. The differences between children, TYAs and older adults may imply differences in aetiology. Information about how current behaviour of children and young people affects their risk of serious illness or death as teenagers and young adults may have a greater influence on personal behaviour than information applicable to diseases that occur in later life.

## Figures and Tables

**Table 1:** Incidence of cancers (rate per million person years at risk) in England, 1979–2001, by age group, cancer group and sex

	**Age group**						**Sex**				**All**	
	**13–14 years**		**15–19 years**		**20–24 years**		**Male**		**Female**			
	******N******	**Rate**	******N******	**Rate**	******N******	**Rate**	******N******	**Rate**	******N******	**Rate**	******N******	**Rate**
Leukaemia	713	24.2	1739	22.8	1483	18.5	2375	25.2	1560	17.1	3935	21.2
Lymphoma	674	22.9	3183	41.7	4641	58.0	4794	50.5	3704	40.0	8498	45.3
CNS neoplasms	771	26.2	1842	24.1	2431	30.4	2671	28.2	2373	25.9	5044	27.1
Bone tumours	434	14.8	1114	14.6	644	8.0	1308	13.9	884	9.8	2192	11.9
Soft tissue sarcoma	191	6.5	703	9.2	810	10.1	930	9.8	774	8.4	1704	9.1
Germ cell tumour	155	5.3	1171	15.3	3370	42.1	4152	43.3	544	6.0	4696	24.8
Melanoma	82	2.8	703	9.2	1995	24.9	967	10.1	1813	19.4	2780	14.7
Carcinoma	264	9.0	1354	17.7	4166	52.0	1658	17.4	4126	44.0	5784	30.6
Misc. NEC	72	2.4	168	2.2	195	2.4	200	2.1	235	2.6	435	2.3
Unclassified	14	0.5	74	1.0	135	1.7	95	1.0	128	1.4	223	1.2
Total	3370	113.6	12051	157.7	19870	248.1	19150	201.5	16141	174.5	35291	188.7

Abbreviation: CNS, central nervous system.

**Table 2:** Incidence of all cancers, leukaemias and lymphomas in teenagers and young adults (13–24 years) in England, 1979–2001, by TDI and GOR

		**Percentage risk ratio by TDI quintile**					******P****-values**					
	**Total cases**	**1 (least)**	**2**	**3**	**4**	**5 (most)**	**Trend**	**Heterogeneity**				
*(a) Case distribution by Townsend deprivation index*												
All cancers	35291	102	102	101	98	96	<0.0001	0.44				
Leukaemia	3935	93	101	96	103	108	0.007	0.43				
ALL	1970	93	105	91	104	107	0.08	0.10				
AML	1371	101	100	103	103	93	0.55	0.69				
CML	325	79	91	86	103	141	0.0005	0.45				
Other leukaemia	269	79	84	102	97	140	0.002	0.58				
Lymphomas	8498	106	101	103	95	95	0.0002	0.36				
NHL	2489	97	98	98	97	110	0.09	0.33				
HL	6009	109	103	106	94	89	<0.0001	0.15				
												
	**Government Office Region**											
**Cancer group**	**North East**	**North West**	**Yorkshire and Humber**	**East Midlands**	**West Midlands**	**East^a^**	**London**	**South East**	**South West**	**All**	******P****-value**	******P**** after TDI**
*(b) Rate per million person years at risk by Government Office Region*												
All cancers	173.1	185.0	185.4	181.2	181.1	177.7	187.1	208.1	208.0	188.7	<0.0001	<0.0001
Leukaemia	21.1	20.7	19.6	20.5	19.6	18.2	24.1	23.3	22.6	21.2	0.0002	<0.0001
ALL	11.1	10.5	9.9	9.9	10.6	9.8	11.3	11.7	11.3	10.7	0.48	0.30
AML	7.4	7.5	6.7	7.3	6.7	5.6	8.2	8.6	7.4	7.4	0.02	0.003
CML	1.9	1.5	1.5	1.7	1.0	1.9	2.4	1.6	2.3	1.7	0.03	0.03
Other leukaemia	0.8	1.2	1.5	1.7	1.3	0.9	2.2	1.4	1.6	1.4	0.010	0.03
Lymphoma	39.9	42.2	42.5	40.7	44.1	46.6	47.6	51.0	48.3	45.3	<0.0001	<0.0001
NHL	12.2	12.1	12.3	11.1	12.9	12.0	15.6	14.7	14.8	13.3	0.0001	0.0001
HL	27.7	30.1	30.2	29.6	31.2	34.5	32.0	36.3	33.5	32.0	<0.0001	0.005

Abbreviations: ALL, acute lymphoblastic leukaemia; AML, acute myeloid leukaemia; CML, chronic myeloid leukaemia; GOR, Government Office Region; HL, Hodgkin lymphoma; NHL, non-Hodgkin lymphoma; TDI, Townsend deprivation index.

a East is the label for the East of England GOR.

**Table 3:** Incidence of CNS neoplasms in teenagers and young adults (13–24 years) in England, 1979–2001, by TDI and GOR

		**Percentage risk ratio by TDI quintile**					******P****-values**					
	**Total cases**	**1 (least)**	**2**	**3**	**4**	**5 (most)**	**Trend**	**Heterogeneity**				
*(a) Case distribution by Townsend deprivation index*												
All CNS neoplasms	5044	104	103	104	94	94	0.003	0.39				
Astrocytoma	1725	104	100	101	96	99	0.45	0.91				
Other gliomas	683	106	96	114	100	83	0.11	0.17				
Ependymoma	264	104	109	97	89	100	0.54	0.84				
PNET	331	95	119	106	80	99	0.43	0.18				
Other specified	1679	105	102	102	97	94	0.12	0.97				
Unspecified	362	113	112	112	78	83	0.01	0.41				
												
	**Government Office Region**											
**Cancer group**	**North East**	**North West**	**Yorkshire and Humber**	**East Midland**	**West Midlands**	**East**	**London**	**South East**	**South West**	**All**	******P****-value**	******P**** after TDI**
*(b) Rate per million person years at risk by Government Office Region*												
All CNS neoplasms	26.0	27.5	28.6	28.4	23.2	23.4	25.2	30.2	30.6	27.1	<0.0001	<0.0001
Astrocytoma	8.8	10.0	10.7	9.2	7.4	7.6	8.4	10.3	10.8	9.3	0.0002	0.0001
Other Gliomas	4.0	3.3	3.5	3.3	2.0	4.1	3.8	4.7	4.0	3.7	0.0003	0.0004
Ependymoma	1.4	1.5	1.2	1.4	1.0	1.2	1.8	1.6	1.2	1.4	0.29	0.19
PNET	1.1	1.9	2.0	2.0	1.3	1.8	1.9	2.1	1.5	1.8	0.24	0.26
Other specified	10.1	9.6	10.2	11.8	5.6	7.5	8.3	9.3	9.5	9.0	<0.0001	<0.0001
Unspecified	0.7	1.1	0.9	0.8	6.0	1.2	0.9	2.2	3.5	1.9	<0.0001	<0.0001

Abbreviations: CNS, central nervous system; GOR, Government Office Region; TDI, Townsend deprivation index.

**Table 4:** Incidence of bone tumours and STS in teenagers and young adults (13–24 years) in England, 1979–2001, by TDI and GOR

		**Percentage risk ratio by TDI quintile**					******P****-values**					
	**Total cases**	**1 (least)**	**2**	**3**	**4**	**5 (most)**	**Trend**	**Heterogeneity**				
*(a) Case distribution by Townsend deprivation index*												
Bone tumours	2192	97	105	100	101	98	0.96	0.62				
Osteosarcoma	1105	93	101	101	102	105	0.21	0.93				
Chondrosarcoma	155	117	96	74	91	122	0.95	0.19				
Ewings tumour	744	96	112	103	102	86	0.30	0.24				
Other bone tumours	188	109	105	107	97	82	0.24	0.93				
Soft tissue sarcomas	1704	97	93	98	108	104	0.09	0.63				
Fibromatous neoplasms	232	84	97	106	114	99	0.29	0.73				
Rhabdomyosarcoma	427	106	90	95	98	111	0.67	0.53				
Other specified STS	704	98	93	92	109	107	0.21	0.61				
STS, NOS	341	91	96	107	111	95	0.51	0.66				
												
	**Government Office Region**											
**Cancer group**	**North East**	**North West**	**Yorkshire and Humber**	**East Midlands**	**West Midlands**	**East**	**London**	**South East**	**South West**	**All**	******P****-value**	******P**** after TDI**
*(b) Rate per million person years at risk by Government Office Region*												
Bone tumours	11.8	11.3	11.8	13.4	11.8	12.0	12.0	12.2	10.9	11.9	0.72	0.71
Osteosarcoma	5.9	6.4	6.3	6.4	4.9	6.1	6.3	6.0	5.4	6.0	0.49	0.46
Chondrosarcoma	1.2	0.6	0.8	1.2	1.0	0.6	0.7	0.7	0.9	0.8	0.42	0.41
Ewings tumour	4.1	3.8	4.1	4.9	2.9	4.6	4.3	4.3	3.5	4.0	0.08	0.08
Other bone tumours	0.6	0.4	0.5	0.8	3.1	0.7	0.7	1.2	1.0	1.0	<0.0001	<0.0001
Soft tissue sarcomas	7.6	7.3	9.5	8.9	8.6	8.8	10.7	10.0	9.5	9.1	0.001	0.0008
Fibromatous neoplasms	1.4	1.0	1.9	1.2	0.7	1.4	1.3	1.1	1.3	1.2	0.05	0.05
Rhabdomyosarcoma	2.0	2.0	2.0	2.5	2.1	2.0	2.1	3.0	2.8	2.3	0.18	0.09
Other specified STS	3.0	2.9	3.8	3.2	3.3	3.8	5.2	3.9	3.6	3.8	0.004	0.004
STS, NOS	1.2	1.3	1.8	2.0	2.5	1.6	2.0	2.0	1.7	1.8	0.09	0.10

Abbreviations: GOR, Government Office Region; STS, soft tissue sarcomas; TDI, Townsend deprivation index.

**Table 5:** Incidence of germ cell tumours and melanoma in teenagers and young adults (13–24 years) in England, 1979–2001, by TDI and GOR

		**Percentage risk ratio by TDI quintile**					******P****-values**					
	**Total cases**	**1 (least)**	**2**	**3**	**4**	**5 (most)**	**Trend**	**Heterogeneity**				
*(a) Case distribution by Townsend deprivation index*												
Germ cell tumours	4696	105	105	100	96	93	0.001	0.91				
Gonadal GCT	4314	107	106	100	96	92	0.0002	0.94				
Non-gonadal GCT	382	91	99	105	93	112	0.30	0.75				
Melanoma	2780	123	116	106	91	68	<0.0001	0.01				
												
	**Government Office Region**											
**Cancer group**	**North East**	**North West**	**Yorkshire and Humber**	**East Midlands**	**West Midlands**	**East**	**London**	**South East**	**South West**	**All**	******P****-value**	******P**** after TDI**
*(b) Rate per million person years at risk by Government Office Region*												
Germ cell tumours	21.5	24.7	24.1	25.4	23.6	25.1	22.4	27.0	29.3	24.8	0.0007	0.05
Gonadal GCT	19.1	22.3	21.9	23.7	21.5	23.0	20.1	25.2	27.5	22.8	<0.0001	0.01
Non-gonadal GCT	2.4	2.3	2.2	1.7	2.1	2.0	2.3	1.8	1.8	2.0	0.74	0.76
Melanoma	13.3	15.0	15.6	12.2	12.7	13.9	11.6	17.8	19.5	14.7	<0.0001	<0.0001

Abbreviations: GCT, germ cell tumours; GOR, Government Office Region; TDI, Townsend deprivation index.

**Table 6:** Incidence of carcinomas in teenagers and young adults (13–24 years) in England, 1979–2001, by TDI and GOR

		**Percentage risk ratio by TDI quintile**					******P****-values**					
	**Total cases**	**1 (least)**	**2**	**3**	**4**	**5 (most)**	**Trend**	**Heterogeneity**				
*(a) Case distribution by Townsend deprivation index*												
Carcinomas	5784	94	98	101	100	105	0.72	0.78				
Thyroid	1131	95	102	102	103	99	0.08	0.85				
Other head and neck	603	88	101	95	103	112	0.09	0.86				
Lung	155	87	89	90	108	125	0.11	0.93				
Breast	489	103	118	93	103	86	0.02	0.33				
Cervix	929	74	92	111	105	111	0.0004	0.16				
Other GU tract	1130	104	96	102	95	103	0.91	0.68				
Colon and rectum	576	107	96	99	87	111	0.98	0.25				
Other GI tract	387	87	89	97	115	112	0.04	0.87				
Other specific and unspecific	384	98	98	105	84	114	0.72	0.30				
												
	**Government Office Region**											
**Cancer group**	**North East**	**North West**	**Yorkshire and Humber**	**East Midlands**	**West Midlands**	**East**	**London**	**South East**	**South West**	**All**	******P****-value**	******P**** after TDI**
*(b) Rate per million person years at risk by Government Office Region*												
Carcinomas	29.3	32.2	30.8	29.1	31.4	26.4	30.1	32.2	31.7	30.6	0.008	0.002
Thyroid	5.5	6.1	6.4	5.6	6.1	6.6	6.3	5.5	5.6	6.0	0.84	0.86
Other head and neck	3.6	3.4	2.3	3.2	3.0	3.0	3.6	3.7	2.8	3.2	0.20	0.17
Lung	0.7	1.0	0.6	0.5	0.8	0.3	0.8	1.0	1.5	0.8	0.002	0.001
Breast	2.6	2.4	2.5	2.4	2.5	1.7	3.0	3.1	2.3	2.6	0.10	0.02
Cervix	10.3	12.6	12.0	8.1	14.0	6.8	6.0	8.6	11.3	9.8	<0.0001	<0.0001
Other GU tract	5.1	5.9	5.3	6.6	5.6	5.5	6.1	6.4	6.6	6.0	0.56	0.62
Colon and rectum	3.1	3.1	2.9	2.9	2.6	1.9	2.6	4.4	3.5	3.1	0.0003	0.0002
Other GI tract	0.9	1.8	2.5	1.7	2.0	2.1	2.3	2.2	2.3	2.1	0.10	0.08
Other specific and unspecific	2.7	2.1	2.2	2.2	1.8	2.2	2.2	1.8	1.7	2.1	0.76	0.59

Abbreviations: GI, gastrointestinal; GOR, Government Office Region; GU, genitourinary; TDI, Townsend deprivation index.
